# Deciphering the Efficacy and Mechanisms of Chinese Herbal Medicine for Diabetic Kidney Disease by Integrating Web-Based Biochemical Databases and Real-World Clinical Data: Retrospective Cohort Study

**DOI:** 10.2196/27614

**Published:** 2021-05-11

**Authors:** Chien-Wei Wu, Hsing-Yu Chen, Ching-Wei Yang, Yu-Chun Chen

**Affiliations:** 1 Division of Chinese Internal and Pediatric Medicine Center for Traditional Chinese Medicine Chang Gung Memorial Hospital Taoyuan Taiwan; 2 School of Traditional Chinese Medicine College of Medicine Chang Gung University Taoyuan Taiwan; 3 Graduate Institute of Clinical Medical Sciences College of Medicine Chang Gung University Taoyuan Taiwan; 4 School of Medicine Faculty of Medicine National Yang Ming Chiao Tung University Taipei Taiwan; 5 Department of Family Medicine Taipei Veterans General Hospital Taipei Taiwan; 6 Institute of Hospital and Health Care Administration National Yang Ming Chiao Tung University Taipei Taiwan

**Keywords:** association rule mining, Chinese medicine network, social network analysis, survival

## Abstract

**Background:**

Diabetic kidney disease (DKD) is one of the most crucial causes of chronic kidney disease (CKD). However, the efficacy and biomedical mechanisms of Chinese herbal medicine (CHM) for DKD in clinical settings remain unclear.

**Objective:**

This study aimed to analyze the outcomes of DKD patients with CHM-only management and the possible molecular pathways of CHM by integrating web-based biomedical databases and real-world clinical data.

**Methods:**

A total of 152,357 patients with incident DKD from 2004 to 2012 were identified from the National Health Insurance Research Database (NHIRD) in Taiwan. The risk of mortality was estimated with the Kaplan-Meier method and Cox regression considering demographic covariates. The inverse probability of treatment weighting was used for confounding bias between CHM users and nonusers. Furthermore, to decipher the CHM used for DKD, we analyzed all CHM prescriptions using the Chinese Herbal Medicine Network (CMN), which combined association rule mining and social network analysis for all CHM prescriptions. Further, web-based biomedical databases, including STITCH, STRING, BindingDB, TCMSP, TCM@Taiwan, and DisGeNET, were integrated with the CMN and commonly used Western medicine (WM) to explore the differences in possible target proteins and molecular pathways between CHM and WM. An application programming interface was used to assess these online databases to obtain the latest biomedical information.

**Results:**

About 13.7% (20,947/131,410) of patients were classified as CHM users among eligible DKD patients. The median follow-up duration of all patients was 2.49 years. The cumulative mortality rate in the CHM cohort was significantly lower than that in the WM cohort (28% vs 48%, *P*<.001). The risk of mortality was 0.41 in the CHM cohort with covariate adjustment (99% CI 0.38-0.43; *P*<.001). A total of 173,525 CHM prescriptions were used to construct the CMN with 11 CHM clusters. CHM covered more DKD-related proteins and pathways than WM; nevertheless, WM aimed at managing DKD more specifically. From the overrepresentation tests carried out by the online website Reactome, the molecular pathways covered by the CHM clusters in the CMN and WM seemed distinctive but complementary. Complementary effects were also found among DKD patients with concurrent WM and CHM use. The risk of mortality for CHM users under renin-angiotensin-aldosterone system (RAAS) inhibition therapy was lower than that for CHM nonusers among DKD patients with hypertension (adjusted hazard ratio [aHR] 0.47, 99% CI 0.45-0.51; *P*<.001), chronic heart failure (aHR 0.43, 99% CI 0.37-0.51; *P*<.001), and ischemic heart disease (aHR 0.46, 99% CI 0.41-0.51; *P*<.001).

**Conclusions:**

CHM users among DKD patients seemed to have a lower risk of mortality, which may benefit from potentially synergistic renoprotection effects. The framework of integrating real-world clinical databases and web-based biomedical databases could help in exploring the roles of treatments for diseases.

## Introduction

Diabetic kidney disease (DKD) is one of the most crucial causes of chronic kidney disease (CKD) and end-stage renal disease (ESRD) at the final disease stage, especially when the prevalence of DKD keeps increasing yearly [1]. It has been reported that about one-third of DKD patients may experience ESRD during their lifetime [2]. Owing to the high prevalence and severe consequences, DKD has become a vital health care problem and causes tremendous financial burden [3-5]. The pathogenesis of diabetic nephropathy is complicated; however, the treatment modalities are still limited and need to be explored. Glomerular hyperfiltration, podocyte dysfunction, basement membrane thickening, mesangial cell proliferation, and collagen deposition with glomerular sclerosis are extensively reported [6-8]. Additionally, several precipitating factors have been identified, including hyperglycemia, advanced glycation end products, activation of the renin-angiotensin-aldosterone system (RAAS), decreased expression of nephrin and integrin, activation of cytokines, profibrotic elements, inflammation, oxidative stress, and vascular growth factors [9-12].

Although there are many Western medicine (WM) options for DKD, only blockade of the RAAS has been identified as an effective treatment, and the agents include angiotensin-converting enzyme inhibitors (ACEis), angiotensin receptor blockers (ARBs), and direct renin inhibitors (DRIs) [13-18]. Several notable novel agents have been recently reported to have benefits for reducing progression to DKD among diabetes mellitus (DM) patients, and these agents include sodium-glucose cotransporter 2 inhibitors (SGLT2is), glucagon-like peptide-1 (GLP-1) agonists, a selective endothelin-1 receptor antagonist, and a nonsteroidal mineralocorticoid receptor antagonist. However, the effectiveness of these agents among DM patients who are already diagnosed with DKD remains unclear, and some clinical trials are ongoing to address these issues [19-22]. Only GLP-1 agonists and SGLT2is have been found to be beneficial in DKD patients [23,24]. These novel agents inspire researchers to study other medications with similar effects on similar pathways and new therapeutic agents for CKD/DKD [16].

Complementary and alternative medicine may be another treatment option to relieve DKD in addition to WM. Several treatment modalities, including Chinese herbal medicine (CHM) and acupuncture, have been reported to have potential therapeutic benefits for DKD [25-28]. Moreover, some medications may be used to relieve proteinuria and ameliorate renal dysfunction, such as *Astragalus membranaceus* (Fisch.) and Liu-Wei-Di-Huang-Wan [29-31]. The potential mechanisms include anti-inflammation, antifibrosis, antioxidation, immunomodulation, and regulation of podocyte dysfunction [30-36]. Besides, some CHMs have been found to have effects on tubular cell cycle modulation [37]. However, only some of the abovementioned herbs/ingredients have been examined in terms of the clinical efficacy in treating DKD, and, on the other hand, only a small proportion of CHMs used in clinical trials have been examined in terms of the possible mechanisms in treating DKD owing to the high heterogeneity in used CHMs for DKD [31,38]. Additionally, the CHM prescriptions used for diseases are usually complicated in the clinical setting, and we previously found that the use of four to five kinds of CHMs in one prescription is not uncommon [39]. A comprehensive summary of the efficacy of CHM prescriptions becomes crucial to understand the effects of CHM for DKD [40,41].

Several methods have been proposed to extract valuable information from complicated CHM prescriptions, such as association rule mining, clustering, and decision tree [42]. In recent years, network pharmacology based on web-based biomedical resources has become one of the most critical tools to analyze CHM prescriptions [43-45]. However, the integration of these techniques with real-world clinical data has been lacking. For DKD, Zhang et al reported the potential effects of six representative compounds in the Gandi capsule (a mixture of several CHMs with fixed proportions) for 99 potential DKD-related target proteins [46]. Moreover, Shi et al tried to use the molecule-protein docking method to predict the possible mechanisms of Bushenhuoxue formula for treating CKD. They identified the potential of tanshinone IIA, rhein, curcumin, calycosin, and quercetin to act on CKD-related proteins, which may be related to the regulation of coagulation and fibrinolytic balance, aberrant extracellular matrix accumulation, and inflammation [47]. However, owing to the lack of clinical data, the effectiveness of these CHM formulae for DKD and the interactions between these CHMs and WMs remain unclear [48]. Besides, the interactions between CHMs and WMs are essential to understand the role of CHM in the modern health care system and the unexpected effects of CHM on DKD from the perspective of molecular medicine. For the above reasons, an integrative analysis on real-world data and web-based biomedical resources with the long-term effects of CHM and synergistic effects of CHM and WM is demanded and necessary for the management of DKD.

In our previous findings, DKD patients who received all kinds of Traditional Chinese medicine (TCM) treatments, including CHM, acupuncture, and moxibustion, had a better prognosis, which raised our interest in CHM use for DKD patients and the possible effective biomedical pathways [41]. In our previous successful integration of the most up-to-date web-based biomedical databases and real-world prescription databases, we identified the synergistic effects of CHM and WM for allergic rhinitis [40]. This study aimed to analyze the outcomes of DKD patients with CHM-only management and elucidate the roles of CHM and WM for DKD using an integrative platform with clinical and web-based biomedical databases.

## Methods

### Data Source and Study Design

The National Health Insurance Research Database (NHIRD), with high coverage (>99%) of all medical records in Taiwan, was used as a prescription data source for this study. The clinical data were preprocessed in our previous reports, including patient demographic features and prescriptions, and the protocol was approved by the Institutional Review Board of Chang Gung Memorial Foundation (number: 103-1259B) [41]. DKD patients were identified with a diagnosis of CKD after DM. From January 1, 2004, to December 31, 2012, DM patients were recognized by using International Classification of Diseases 9, Clinical Modification (ICD-9-CM) codes 250.0-250.9 and antidiabetic medications, including insulin and biguanides sulfonylurea, an alpha-glucosidase inhibitor, thiazolidinediones, and DDP-4 inhibitors. Furthermore, CKD was recognized by using ICD-9-CM codes 580.X-588.X, 250.4x, 274.1x, 283.11, 403.x1, 404.x2, 404.x3, 440.1, 442.1, 447.3, 572.4, 642.1x, and 646.2x. To recognize incident DKD patients, any subjects with previous CKD records or renal transplantation were excluded. Additionally, any visits with the use of acupuncture, massage, or other TCM modalities were excluded. In Taiwan, the diagnosis of DKD is consistent with the guidelines, and detection of diabetic nephropathy (DN) subjects by ICD-9-CM codes was consistent with previous studies [29,41]. CHM users were defined as DN patients who used CHM at least twice for DN from 2004 to 2012, and all CHM prescriptions were collected to build up the Chinese Herbal Medicine Network (CMN) with the integration of web-based biomedical databases.

### Bias Assessment

This data set was unique and particularly suitable for CHM prescription analysis owing to its high coverage of Taiwan’s general population and unbiased selection of CHM as a treatment option [39,49]. Possible selection bias and referral bias could be avoided as much as possible with a nationwide database than with a hospital-based database [50]. Additionally, the exclusion of acupuncture, moxibustion, or manual therapy is helpful to avoid confounding bias with possible influence on CHM prescriptions. Moreover, because there is no recommendation for initiation of CHM treatments, we found that the mean interval from diagnosis of DKD to initiation of CHM use was about 240 days among CHM users (data not shown), and immortal time bias may occur [51,52]. To overcome this problem, a 1-year landmark design was used to avoid the potential immortal time bias. Thus, the study index date was set as 1 year after DKD diagnosis for each patient, and patients who died within 1 year after DKD diagnosis were excluded as well. Moreover, to eliminate the possible baseline differences between CHM users and nonusers, inverse probability treatment weighting (IPTW) according to all assessable covariates described below was used [53].

### Study Covariates and Outcome

Patient gender, age, comorbidities, medications, prior experience of CHM use, geolocation, and insured level were used as covariates in this study. The Charlson comorbidity index (CCI) and Diabetes Complications Severity Index (DCSI), with reduction of two factors (albuminuria and serum creatine) as a modification, were calculated as a summary of DKD-related comorbidities [54,55]. The identification of specific comorbidities was based on ICD-9-CM codes for diseases, including cerebrovascular disease (ICD-9-CM codes 430-432 and 433-435), heart failure (ICD-9-CM code 428), ischemic heart disease (IHD; ICD-9-CM codes 411, 413, and 414), hypertension (ICD‐9‐CM codes 401‐405), and hyperlipidemia (ICD‐9‐CM code 272). Only patients with at least two diagnosis codes in the outpatient service or one during the hospitalization 1 year before the DKD diagnosis were confirmed as having comorbidities. We also analyzed medications, including insulin; other antihyperglycemic agents; antihypertensive agents; antilipid agents; RAAS blockers, including ACEis, ARBs, and DRIs; aspirin; and nonsteroidal anti-inflammatory drugs (NSAIDs). Only medications with a cumulative duration of more than 30 days were included in the analysis. All-cause mortality was the outcome of this study, and it was recognized when patients permanently withdrew from the insurance program [56,57]. All enrolled DKD patients were followed up from the DKD starting point to the endpoint or the end of 2012.

### CHM Prescriptions in the Database

Traditionally, the medicines used by TCM doctors include not only herbal plants, but also insects, animals, and minerals. In this study, we collectively referred to all medicines recorded in the database as CHM. There are two kinds of CHMs used in clinical practice, namely herbal formula (HF) and single herb (SH). SH is the extract or crude powder of a part of a herbal plant, insect, animal, or mineral and is made following ancient classics’ process methods. On the other hand, HF is composed of more than one SH with the same proportion as recorded in TCM classics and is premixed in the pharmaceutical factory before marketing. More than 600 kinds of SHs and HFs are available for TCM doctors to choose freely, and all SHs and HFs are manufactured in a Good Manufacturing Practice pharmaceutical factory with strict regulation regarding the concentrations of heavy metals and pesticides.

### Statistical Analysis: Outcome Evaluation and Online Pathway Analysis on the CMN

The first part of the statistical analysis was survival analysis. Descriptive statistics were used for CHM users’ demographic characteristics, such as age, gender, comorbidities, insured level, living locations, previous medical use, and prescribing patterns. After applying IPTW to balance the differences between CHM users and nonusers, survival analysis was carried out by performing a Kaplan-Meier estimation with the log-rank test. Additionally, Cox regression with adjustment of the abovementioned assessable covariates was used to estimate the adjusted hazard ratio (aHR) for CHM users. Furthermore, to ensure the analysis results, subgroup analysis was conducted based on age, gender, and comorbidities. Sensitivity tests were also performed with 1:1 matching on CHM users and nonusers, and different study populations.

Second, CHM prescription analysis with integration of the CMN and online biomedical databases was performed to reveal the potential molecular pathways of CHM for DKD. In this step, the application programming interface (API) was used to assess the biomedical databases to obtain the latest information about WM and CHM. As described in our previous studies about CHM prescription analysis, the CMN was constructed by applying association rule mining (ARM) and social network analysis (SNA) on CHM prescriptions for DKD [39,58,59]. Briefly, ARM on CHM prescriptions used for DKD could find out the CHM-CHM combinations commonly used for DKD. These CHM combinations could be connected to form the CMN for DKD, and SNA with these combinations could put CHMs used concurrently into the same cluster. CHM indications acquired from the Chinese Pharmacopoeia (2015 edition) were used to summarize the CHM clusters from TCM viewpoints [39]. On the other hand, four types of WMs used for DKD were proposed in this study, including ACEis, ARBs, GLP-1 agonists, and SGLT2is. Other possible molecular pathways could be obtained based on these CHM clusters and WMs by connecting WMs and CHM clusters to online biomedical databases [40]. Since biomedical databases contained only information about SHs, every HF in the CMN was disassembled to SHs according to the compositions provided by the Department of Chinese Medicine and Pharmacy of the Ministry of Health and Welfare, Taiwan [60]. Next, the ingredients of each SH were obtained from TCMSP [61], TCM-ID [62], and TCM@Taiwan [63], and the information was cross-validated with the Chinese Pharmacopoeia (2015 edition). Each ingredient’s characteristics were also acquired from PubChem, such as oral bioavailability, XlogP, drug likeness, molecular weight, topological polar surface area (TPSA), and simplified molecular input line entry specification (SMILES). This information was crucial to realize the similarities between the ingredients of WMs and CHMs [48].

Moreover, to acquire each ingredient’s possible target proteins for both WM and CHM, the Search Tool for Interacting Chemicals (STITCH) [64,65] was queried. STITCH is a well-developed database composed of known and predicted connections between a chemical compound and target proteins derived from genomic context predictions, high-throughput lab experiments, gene coexpression databases, text mining in journal databases, and previous knowledge from other databases [66]. Up to January 2021, STITCH contained 9,643,763 proteins, 2,031 organisms, and over 430,000 chemical compounds as ingredients in CHMs. Inferred chemical-target protein connections from experiments involving species other than humans and a scoring system to describe the confidence of the connections in this database could be used to explore the connections between chemical compounds and target proteins. The scoring system, ranging from 0 to 1, summarizes the probability of connection occurrence by combining the probability from individual data sources, such as experiments from mice and text mining from journal databases, in a native Bayesian fashion. A higher score symbolizes more substantial confidence in the connection between chemical compounds and target proteins. To select the most confident connections between drug ingredients and target proteins, a threshold of 0.950 was considered.

Furthermore, to assess the molecular pathways for CHMs in the CMN and WMs, the target proteins were sent to the Reactome pathway database via API, where overrepresentation tests were performed to disclose the potential acting pathways of CHMs and WMs [67-69]. Reactome is a freely accessible web resource to estimate, interpret, and visualize the molecular pathways of given groups of genes or proteins. A total of 15 species pathways were included in the Reactome pathway database, and there were 10,929 proteins, 13,534 reactions, and 2477 pathways for humans (last assessed date: January 2020). The overrepresentation analysis was carried out on the hypothesis that if a molecular pathway is relevant, the pathway’s proteins should be more than randomly expected. The false discovery rate (FDR), calculated using the Benjamini-Hochberg approach, was used to demonstrate each pathway’s statistical significance. Pathways with an FDR ≤0.05 were considered.

**Figure 1:**
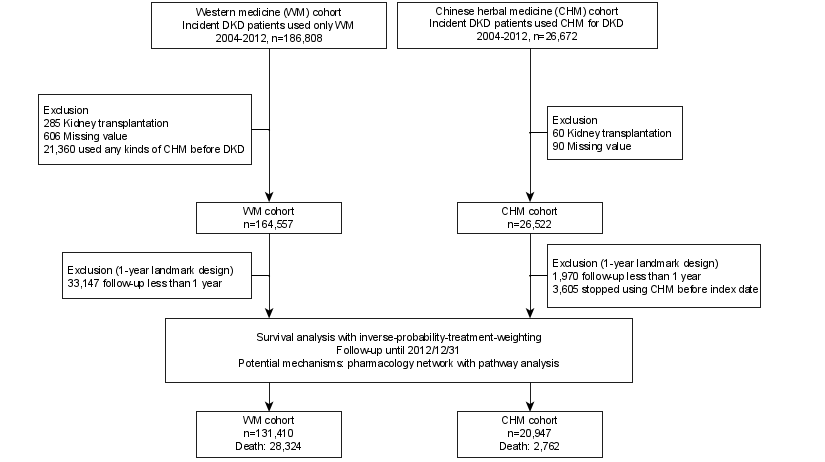
Flow diagram. DKD: diabetic kidney disease.

**Figure 2:**
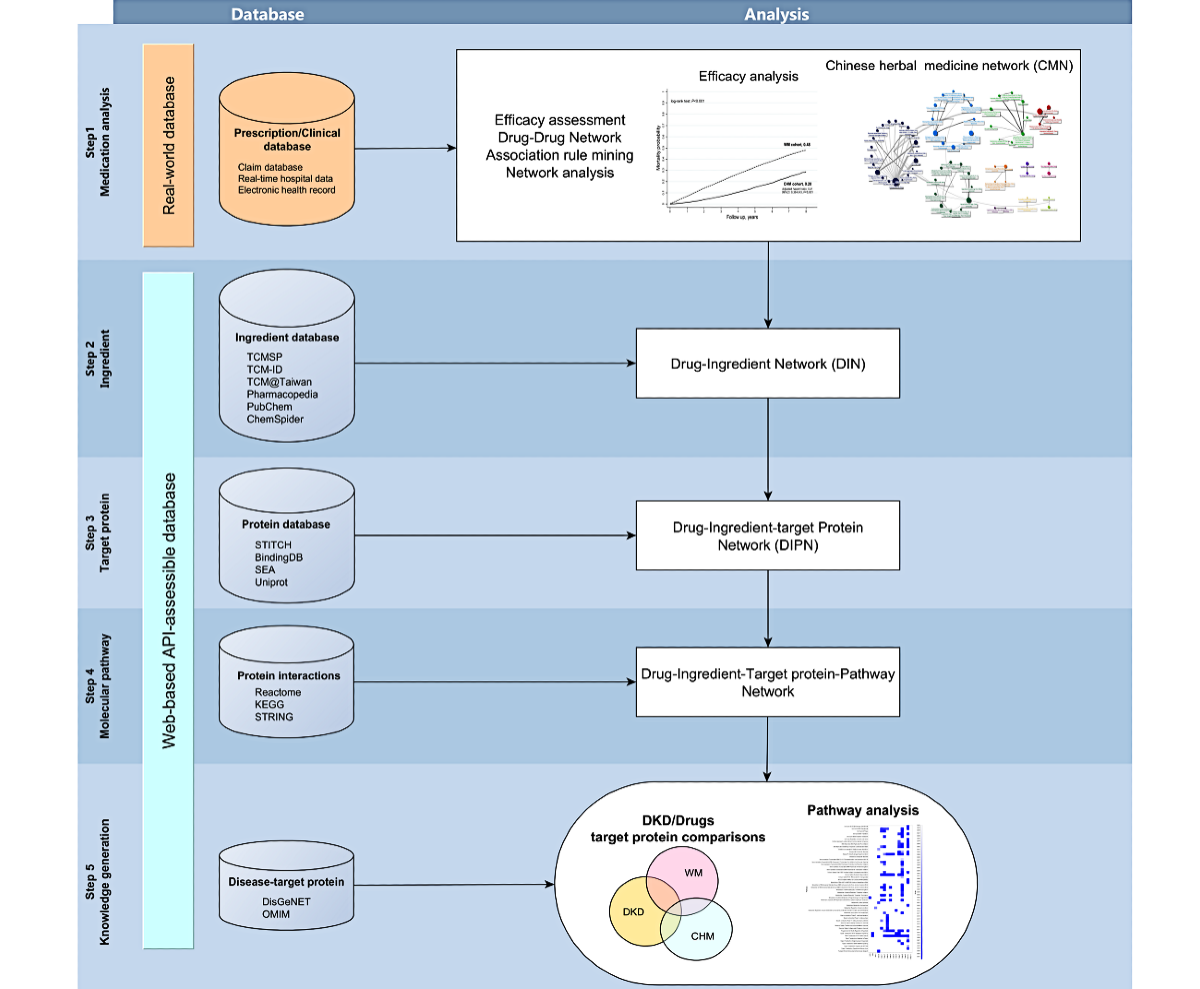
Data processing framework. API: application programming interface; DKD: diabetic kidney disease.

Figures 1 and 2 demonstrate the data processing flow of this study. The freeware KNIME (version 4.0) was used to deal with the clinical and web-based biomedical databases. NodeXL was used to build up the networks and perform SNA [70]. The commercial statistical software STATA (Release 16, StataCorp) was used to carry out survival analysis on core CHMs. A *P* value ≤.05 was considered significant.

## Results

### Baseline Characteristics of CHM Users Among DKD Patients

Table 1 shows the demographic features of the CHM and WM cohorts. Except for the use of cyclooxygenase-2 inhibitors, these two cohorts were quite different. DKD patients who were female, aged 41 to 60 years, lived in urban areas, and had higher income were more commonly seen among CHM users. Regarding underlying diseases, less DKD-related comorbidities, such as hypertension, hyperlipidemia, heart failure, IHD, and cerebrovascular disease, and DM-related complications were found among CHM users. Regarding medications, except NSAIDs and acetaminophen, drugs to control hypertension, IHD, heart failure, hyperlipidemia, and DM were more commonly seen in the WM cohort. On applying the IPTW method, the baseline demographic features of the CHM and WM cohorts were well balanced (all standardized mean differences were within 10% among the CHM and WM cohorts; Multimedia Appendix 1).

**Table 1:** Characteristics of the Chinese herbal medicine and Western medicine cohorts among incident diabetic kidney disease patients from 2004 to 2012.

Characteristic				CHM^a^ cohort (n=20,947), n (%) or mean (SD)		WM^b^ cohort (n=131,410), n (%) or mean (SD)		*P* value
**Gender**								<.001
	Female		9298 (44.4%)		55,546 (42.3%)			
	Male		11,649 (55.6%)		75,864 (57.7%)			
**Age (years)**								<.001
	≤40		991 (4.7%)		4437 (3.4%)			
	41-60		9388 (44.8%)		41,605 (31.7%)			
	≥61		10,568 (50.5%)		85,368 (65.0%)			
**Comorbidities**								
	Hypertension		12,489 (59.6%)		89,219 (67.9%)		<.001	
	Hyperlipidemia		7748 (37.0%)		52,805 (40.2%)		<.001	
	Heart failure		1023 (4.9%)		10,053 (7.7%)		<.001	
	IHD^c^		3674 (17.5%)		24,820 (18.9%)		<.001	
	CVD^d^		1379 (6.6%)		12,765 (9.7%)		<.001	
	Hyperuricemia		1999 (9.5%)		14,477 (11.0%)		<.001	
	Modified DCSI^e^ score, mean (SD)		3.3 (1.2)		3.4 (1.2)		<.001	
**Medications**								
	**Diabetic drugs**							
		Insulin analogs	2674 (12.8%)		23,197 (17.7%)		<.001	
		OHAs^f^	14,487 (69.2%)		97,077 (73.9%)		<.001	
	**Lipid-lowering agents**							
		Statin/fibrate	8139 (38.9%)		57,001 (43.4%)		<.001	
	**Antihypertensives**							
		ACEi^g^/ARB^h^	10,820 (51.7%)		79,763 (60.7%)		<.001	
		Others	15,443 (73.7%)		99,808 (76.0%)		<.001	
	**Analgesics/aspirin**							
		NSAIDs^i^	6783 (32.4%)		33,540 (25.5%)		<.001	
		COX-2^j^ inhibitors	1576 (7.5%)		9931 (7.6%)		0.86	
		Acetaminophen	5767 (27.5%)		28,702 (21.8%)		<.001	
		Aspirin	6356 (30.3%)		43,116 (32.8%)		<.001	
**Insured level (NTD^k,l^/month)**								<.001
	0-20,000		15,802 (75.0%)		105,987 (81.0%)			
	20,001-40,000		3441 (16.0%)		17,366 (13.0%)			
	≥40,001		1704 (8.0%)		8057 (6.0%)			
**Geolocation**								<.001
	Urban		15,437 (73.7%)		92,967 (70.7%)			
	Rural		5510 (26.3%)		38,443 (29.3%)			
Previous TCM^m^ users				11,161 (53.3%)		0 (0.0%)		<.001

^a^CHM: Chinese herbal medicine.

^b^WM: Western medicine.

^c^IHD: ischemic heart disease.

^d^CVD: cerebral vascular disease.

^e^DCSI: Diabetes Complications Severity Index.

^f^OHA: oral hypoglycemic agents.

^g^ACEi: angiotensin-converting enzyme inhibitor.

^h^ARB: angiotensin receptor blocker.

^i^NSAID: nonsteroidal anti-inflammatory drug.

^j^COX-2: cyclooxygenase-2.

^k^NTD: new Taiwan dollar.

^l^1 NTD=0.033 USD.

^m^TCM: traditional Chinese medicine.

### Risk of Mortality Among CHM Users

**Figure 3:**
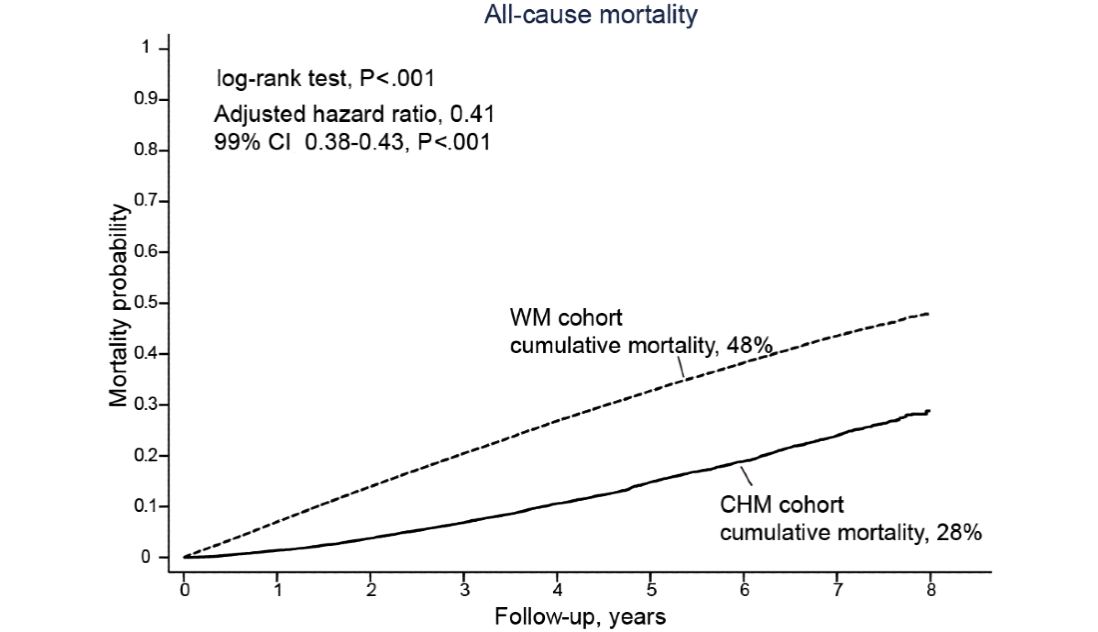
Overall survival benefit among patients using Chinese herbal medicine (CHM) for diabetic kidney disease. Kaplan-Meier curves by patient groups. WM: Western medicine.

At the end of 2012, the median follow-up duration of all patients was 2.49 years, and the cumulative mortality among the CHM cohort was significantly lower than the WM cohort (28% vs 48%, *P*<.001; Figure 3). On adjusting age, gender, socioeconomic status, comorbidities, DM-associated complications, and medications, the risk of mortality was 0.41 among the CHM cohort (99% CI 0.38-0.43; *P*<.001). Furthermore, it seemed that prolonged use of CHM for DKD is safe. The risk of mortality reduced as the duration of CHM increased; DKD patients with CHM use less than 180 days had twice the risk of mortality than patients with CHM use more than 180 days (aHR 0.51 vs 0.25; both *P*<.001 compared to the WM cohort; Table 2). The sensitivity tests including propensity scores with 1:1 matching and the CHM cohort without late users demonstrated similar results (Multimedia Appendix 2). Moreover, when considering the influence of ESRD on DKD, patients in the CHM cohort had lower risk of mortality on either excluding ESRD patients (aHR 0.38, 99% CI 0.36-0.41; *P*<.001) or including ESRD patients (aHR 0.45, 99% CI 0.41-0.50; *P*<.001) (Multimedia Appendix 2). Moreover, the subgroup analysis on mortality risks using multivariate Cox regression showed reduced risks among CHM users stratified by age, gender, and comorbidities.

**Table 2:** Association of Chinese herbal medicine for diabetic kidney disease with lower mortality probability.

Variable			Subjects, n	Deaths, n		aHR^a^		99% CI		*P* value
WM cohort			131,410	28,324		1 (reference)		N/A^b^		N/A
**CHM duration**										
	<180 days	13,670		2093	0.51		0.48-0.54		<.001	
	≥180 days	7277		669	0.25		0.22-0.27		<.001	

^a^aHR: adjusted hazard ratio.

^b^N/A: not applicable.

### CMN for DKD

**Figure 4:**
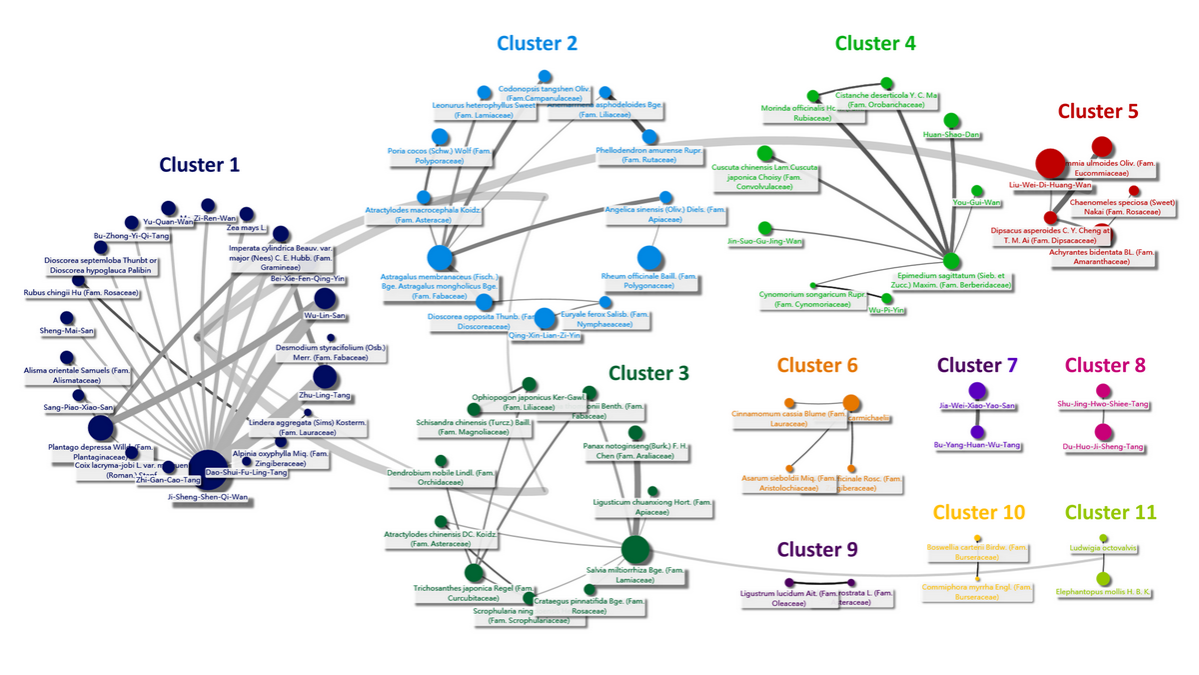
Chinese Herbal Medicine Network (CMN) for diabetic kidney disease. Relations are indicated by grey lines connected to the center of clusters.

A total of 173,525 CHM prescriptions were analyzed during the study period. There were 661 kinds of CHMs used (69.5% of all kinds of CHMs available in Taiwan), and about 5.7 CHMs were used in each prescription on average. Ji-Sheng-Shen-Qi-Wan was used most commonly (22.9%) (Multimedia Appendix 3). Figure 4 demonstrates the CMN for DKD, which was constructed by summarizing the CHM-CHM combinations selected by the ARM from all CHM prescriptions (the top 10 combinations listed in Multimedia Appendix 4). By using SNA to assemble the CHMs commonly used together, a total of 11 clusters could be defined, and the CHMs contained in each cluster are listed in Multimedia Appendix 5. A higher resolution of the CMN graph is provided online as well [71]. The within-cluster CHMs had closer relations than CHMs between clusters, which meant the CHMs in the same cluster were more commonly coprescribed. The network also revealed that other intracluster CHMs frequently connected some CHMs among clusters composed of more than two CHMs, such as Ji-Sheng-Shen-Qi-Wan in cluster 1, *Astragalus membranaceus* (Fisch.) Bge. in cluster 2, *Salvia miltiorrhiza* Bge. in cluster 3, *Epimedium sagittatum* (Sieb. et Zucc.) Maxim. in cluster 4, *Dipsacus asperoides* C. Y. Cheng at T. M. Ai in cluster 5, and *Aconitum carmichaelii* in cluster 6. In their clusters, other CHMs seemed to have to be used with these CHMs as adjuvants. Moreover, some between-cluster relations could be found as well (Figure 4), such as cluster 1-cluster 2, cluster 1-cluster 5, cluster 2-cluster 3, cluster 1-cluster 3, and cluster 1-cluster 11, which adequately represent the complexity of CHM prescriptions in the clinical setting. Taking these prescription patterns together, when dealing with DKD, TCM doctors may use CHM combinations in the same cluster and sometimes more than one cluster. The potential effects of each cluster were assessed, and the trends of reduced risks of mortality were similar among each cluster compared to CHM nonusers (Table 3).

**Table 3:** Cox regressions for mortality and 1-year landmark analysis among Chinese herbal medicine and Western medicine cohorts.

Cluster^a^	Unadjusted				Adjusted^b^		
	HR^c,d^	99% CI	*P* value	aHR^e,f^		99% CI	*P* value
Cluster 1 (n=5272)	0.39	0.35-0.43	<.001	0.40		0.36-0.44	<.001
Cluster 2 (n=2275)	0.37	0.32-0.43	<.001	0.37		0.32-0.44	<.001
Cluster 3 (n=2139)	0.38	0.33-0.45	<.001	0.39		0.34-0.46	<.001
Cluster 4 (n=905)	0.23	0.15-0.36	<.001	0.24		0.16-0.35	<.001
Cluster 5 (n=1144)	0.33	0.26-0.43	<.001	0.34		0.27-0.44	<.001
Cluster 6 (n=665)	0.38	0.28-0.50	<.001	0.38		0.29-0.51	<.001
Cluster 7 (n=173)	0.43	0.25-0.74	<.001	0.48		0.30-0.76	<.001
Cluster 8 (n=375)	0.22	0.10-0.49	<.001	0.23		0.11-0.47	<.001
Cluster 9 (n=243)	0.32	0.19-0.54	<.001	0.32		0.20-0.50	<.001
Cluster 10 (n=286)	0.33	0.20-0.53	<.001	0.32		0.21-0.48	<.001
Cluster 11 (n=196)	0.45	0.27-0.73	<.001	0.45		0.29-0.69	<.001

^a^Each cluster contained patients who took different groups of Chinese herbal medicines.

^b^Gender, age, geolocation, insured level, comorbidities, and medications were used as covariates in the adjusted regression models.

^c^HR: hazard ratio.

^d^The hazard ratio of each cluster was estimated after inverse probability treatment weighting in contrast to the Western medicine cohort.

^e^aHR: adjusted hazard ratio.

^f^The adjusted hazard ratio was calculated by a Cox regression model considering patient gender, age, comorbidities, medications, insured level, and geolocation. Inverse probability treatment weighting was estimated from the same covariates to relieve the accessible confounding bias between Chinese herbal medicine users and nonusers.

### Web-Based Molecular Pathway Exploration Regarding CHM Clusters and WMs

**Figure 5:**
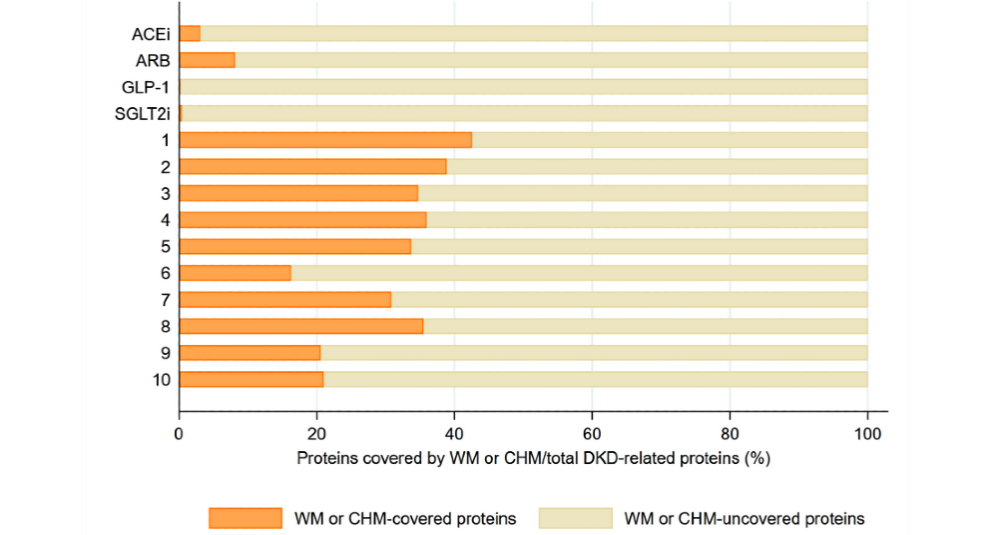
The proportion of DKD-related proteins covered by WM and CHM. A higher proportion of DKD-related proteins is covered by CHM clusters. ACEi: angiotensin-converting enzyme inhibitor; ARB: angiotensin receptor blocker; CHM: Chinese herbal medicine; DKD: diabetic kidney disease; GLP-1: glucagon-like peptide-1; SGLT2i: sodium-glucose cotransporter 2 inhibitors; WM: Western medicine.

**Figure 6:**
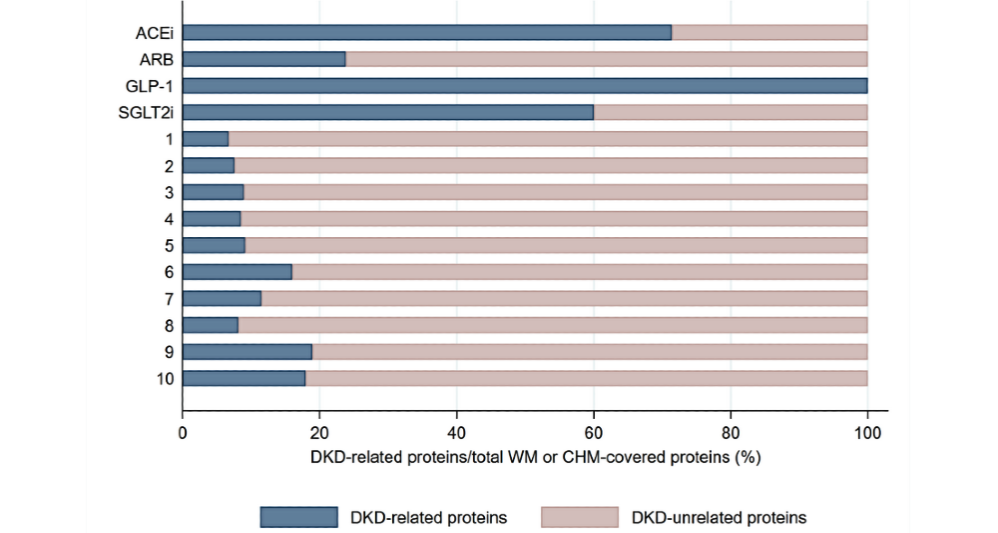
The proportion of proteins covered by CHM or WM specific to DKD. WM aimed more specifically at DKD-related proteins. ACEi: angiotensin-converting enzyme inhibitor; ARB: angiotensin receptor blocker; CHM: Chinese herbal medicine; DKD: diabetic kidney disease; GLP-1: glucagon-like peptide-1; SGLT2i: sodium-glucose cotransporter 2 inhibitors; WM: Western medicine.

Figure 5 shows the associations between DKD-related proteins and CHM clusters or WMs on searching potential target proteins for clinically commonly used CHMs and WMs in a web-based database as mentioned above. The examples of connections between CHMs, CHM ingredients, and target proteins are listed in Multimedia Appendix 6. There were 767 ingredients contained in CHMs in the CMN and 37 WMs in four types of WMs. The physiochemical characteristics of CHMs and WMs were quite different (Multimedia Appendix 7 and Multimedia Appendix 8). Figure 5 reveals CHM clusters often covering more DKD-related proteins than WMs commonly used for DKD; however, we also found that CHM clusters often covered much more DKD-unrelated target proteins than WMs (Figure 6).

**Figure 7:**
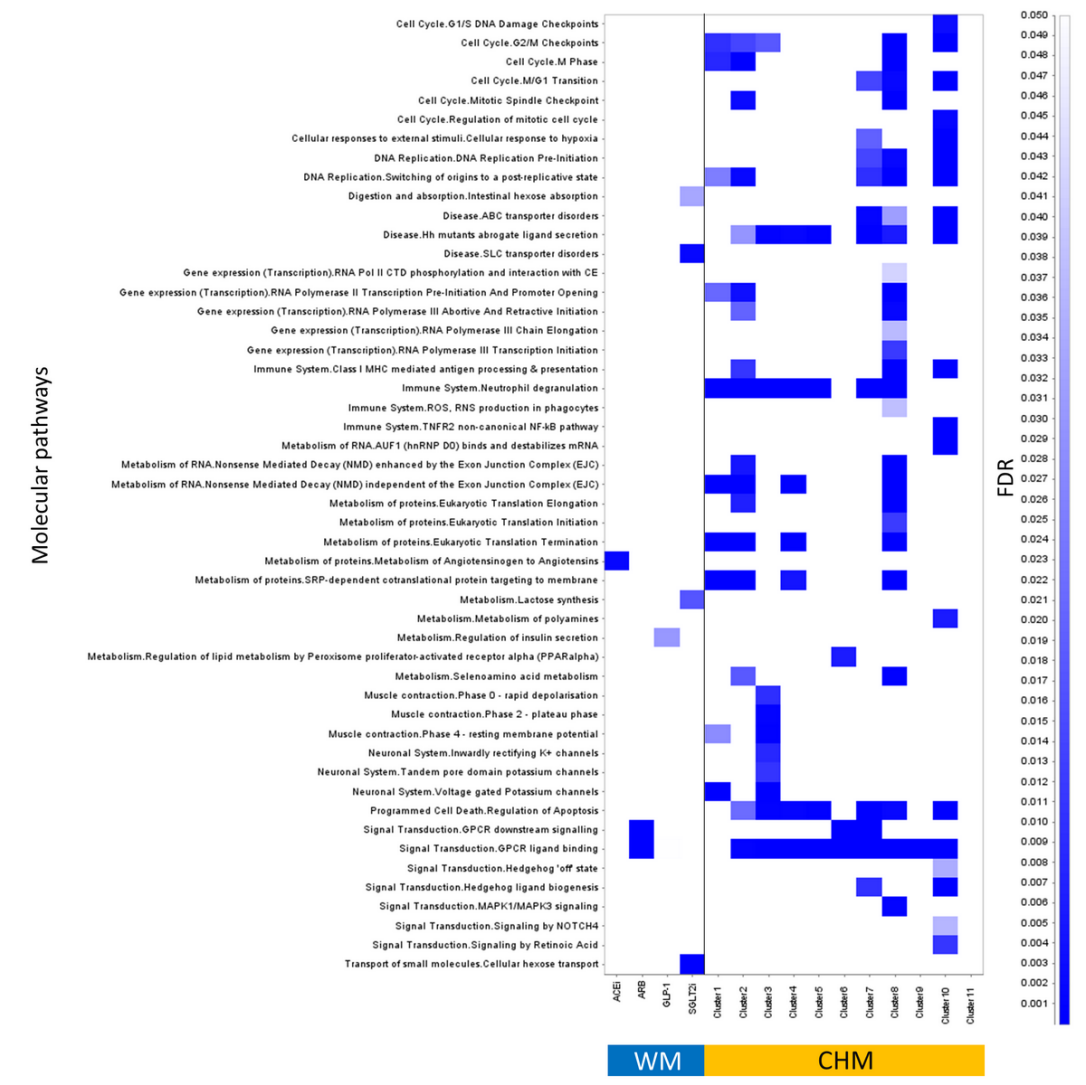
Summary of biomedical pathways covered by clusters of Chinese herbal medicine (CHM) and Western medicine (WM). Pathway overrepresentation analysis and classification was performed by assessing the online Reactome database, and only pathways with a false discovery rate ≤0.05 were considered.

Moreover, it was notable that Figure 7 shows the diverse molecular pathways covered by CHM clusters and WMs. CHM clusters potentially covered more pathways than WMs. The pathways in CHM clusters, which include GPCR ligand blinding and GPCR downstream signaling, overlapped with ARB pathways. On the contrary, pathways of ACEis, GLP-1 agonists, and SGLT2is had no intersection with CHM clusters. Moreover, many CHM cluster pathways were not covered by WMs, such as cell cycle, gene regulation, and metabolism pathways. Table 4 shows the possibly complementary effects of WMs and CHMs, since their molecular pathways seemed rather distinctive. DKD patients with hypertension, HF, and IHD who used RAAS blockers and CHMs had lower risks of mortality than those who used RAAS blockers alone (aHR 0.47, 99% CI 0.45-0.51; *P*<.001; aHR 0.43, 99% CI 0.37-0.51; *P*<.001; and aHR 0.46, 99% CI 0.41-0.51; *P*<.001, respectively).

**Table 4:** Risks of mortality among Chinese herbal medicine users with chronic heart failure, hypertension, and ischemic heart disease under renin-angiotensin-aldosterone system inhibition therapy.

Disease in patients who received renin-angiotensin-aldosterone system inhibition blockers	CHM^a^ nonusers		CHM users		aHR^b,c^	99% CI	*P* value
	Events, n	Subjects, n	Events, n	Subjects, n			
Hypertension	18,310	78,935	1813	10,814	0.47	0.45-0.51	<.001
Chronic heart failure	3876	9369	269	977	0.43	0.37-0.51	<.001
Ischemic heart disease	6408	21,359	600	2967	0.46	0.41-0.51	<.001
Hypertension, chronic heart failure, or ischemic heart disease	19,225	82,110	1919	11,240	0.48	0.45-0.51	<.001

^a^CHM: Chinese herbal medicine.

^b^aHR: adjusted hazard ratio.

^c^The aHR was calculated by a Cox regression model considering patient gender, age, comorbidities, medications, insured level, and geolocation. Inverse probability treatment weighting was estimated from the same covariates to relieve the accessible confounding bias between Chinese herbal medicine users and nonusers.

## Discussion

### Principal Findings

This is the first study to analyze CHM prescriptions for incident DKD patients. Potential survival benefits and molecular pathways present the potential complementary roles of CHMs in managing DKD. We previously proposed a framework to connect clinical databases to web-based biochemical and pathway databases to predict the efficacy of using CHM, and we reported the possible acting pathways of CHM for DKD with the same framework [40]. The different pathways among CHM and WM may have synergistic effects for DKD. By integrating web-based biomedical databases with the CMN, we found several clusters after analyzing the common use of CHM in the NHIRD, which reflected the TCM viewpoints and prescription patterns for DKD. The lower mortality risks among CHM clusters for DKD revealed the potential usefulness of CHM among DKD patients.

Most importantly, by using the web-based Reactome pathway database, the pathways of CHM could be comprehensively overviewed. There are many pathways covered by CHM clusters, but which are not seen in WM. Furthermore, the complementary effects could be validated by clinical data. The framework of cross-utilization of clinical and web-based biochemical databases showed the possibility to decipher CHM treatments for diseases.

Antihypertensive medicines, such as ACEis and ARBs, are recommended in patients with DKD. They are proven to reduce mortality rates and prevent cardiovascular morbidity. In addition, they can slow the degeneration of kidney function in patients with hyperalbuminuria and pre-ESRD [72-74]. Some studies have declared that simultaneous use of more than one drug is a good strategy for treating DKD patients because of different mechanisms [75-77]. However, whether CHM should be used with ACEis or ARBs remained unexplored, even though use of CHM with ACEis or ARBs simultaneously may improve blood pressure control among hypertension patients [78]. Our study reported the rationale of combining an ACEi or ARB with CHM for DKD patients by presenting the long-term benefits and the different coverage of DKD-related proteins and molecular pathways. Notably, CHM clusters used for DKD often cover more DKD-related proteins and pathways than WMs. Most pathways overrepresented by CHM are different from those related to ACEis or ARBs, such as cell cycle and gene regulation.

Cell cycle and gene regulation seemed to be the most different covered pathways between CHM and WM. A previous study found that specific CHMs are involved in DKD-related modulation of microRNA [79]. Besides, the importance of cell cycle arrest in treating CKD seems to be increasing in recent years [80-84]. Cell division involves the following four phases: G0-G1, S, G2, and M. To repair the injured tissue ultimately, DNA is replicated and divided in the process of the cell cycle. Checkpoints play an important role in the quality assurance process during cell division [85]. It is reported that proximal tubular cells arrested in the G2/M phase after an injury are responsible for the fibrotic response, which leads to CKD [83,84,86]. Hence, helping cells abrogate the G2/M arrest and preventing profibrotic growth factor release are new strategies for avoiding renal fibrosis [81,87]. According to our results, several pathways related to the cell cycle may be covered by CHM clusters, especially in pathways related to G2/M checkpoints. With probable regulation in the cell cycle, CHM may target specific sites and participate in cellular repair. Owing to multiple targets in CHM, the simultaneous use of CHM and WM provides a complementary treatment and another perspective in patients with DKD under ACEi or ARB therapy.

In addition to the pathways proposed by integrating biomedical databases with clinical databases, the CMN also revealed TCM viewpoints on managing DKD. TCM doctors categorize diseases into different specific patterns according to the patients’ symptoms and clinical conditions. This characteristic approach to personalized diagnosis and treatment is named “bian-zheng-lun-zhi.” In our study, 11 clusters according to the frequency and coprescription of CHMs were classified. These clusters with specific features explain that “bian-zheng-lun-zhi” has meticulous assessment and better outcomes. Ji-Sheng-Shen-Qi-Wan, which was used most commonly in our study, is the prescription to treat a patient with DKD diagnosed as having “Kidney-Yang Deficiency” in TCM. In TCM theory, “Kidney-Yang” indicates that the functions of the reproductive, endocrine, and urinary systems are normal, and “Kidney-Yang Deficiency” indicates hypofunctioning of these systems [88]. Several molecular pathways, such as cell cycle, gene regulation, signal transduction, immune system, and metabolism, were found to be involved in “Kidney-Yang” [89,90]. These pathways are similar to the pathways in which Ji-Sheng-Shen-Qi-Wan in cluster 1 is overrepresented. Therefore, the associations of DKD-related mechanisms and specific patterns of TCM are revealed by this framework.

Safety is one of the outcomes that cannot be ignored. Specific CHMs have been banned because they were proven to cause damage to the kidney, such as aristolochic acid–containing herbs [49,91]. We used the cohort from 2004 to exclude any potential adverse effects of aristolochic acid–containing herbs, and it helped us evaluate patient outcomes when using CHMs. Furthermore, we proved that using CHMs might be safe and beneficial for DKD patients in the long term. Our study indicates that more prolonged use of CHM among DKD patients reduces the mortality rate, especially in DKD patients who use CHM for more than 180 days.

### Limitations

There are some limitations in this study. First, the severity and stage of DKD could not be analyzed owing to the lack of clinical information, such as glycemia, blood pressure, complete blood count, and biochemistry. The actual quality of control in DM and hypertension is crucial to patients with DKD. While this study aimed to compare mortality between WM and CHM, and explore the use of CHM, quite a few patients used CHM for nonfatal conditions that involved serious effects on quality of life, such as diabetic ophthalmopathy and limb necrosis. Future studies should focus on comparisons in such nonfatal conditions. Second, data about self-paid CHM and folk medicine were absent because only reimbursed CHM treatments were included. In Taiwan, most CHM treatments are fully reimbursed and convenient. Therefore, our study results would not be greatly affected by the use of self-paid CHM and folk medicine. Third, new oral hypoglycemic agents, such as SGLT2is, were not included in the analysis. These agents have the advantage of lowering mortality rates and cardiovascular morbidity among DKD patients [77]. Although they were not included in this study owing to approval in Taiwan in 2014, more studies about combined therapy involving SGLT2is are important in the future, since the pathways covered by SGLT2is were found to be quite different in our study.

### Conclusion

By integrating clinical and biomedical databases, lower mortality rates among CHM users were found, and the complementary roles of CHM and WM may be the reason. Since CHM has complementary effects and proven safety, it may be beneficial to consider TCM treatment in DKD patients under WM therapy. Our study’s main advantage is the clarification of the summary of the mechanisms of CHM for DKD in the real world, which may broaden the horizon for DKD and facilitate the development of new drugs from active ingredients in CHM. Further studies, including those involving more detailed information about patients’ conditions and analysis of prescribed CHMs, are required.

## Appendix

Multimedia Appendix 1 Standardized mean differences (%) after inverse probability treatment weighting between Chinese herbal medicine clusters and the Western medicine cohort.

Multimedia Appendix 2 Sensitivity analyses on the risks of mortality among Chinese herbal medicine users.

Multimedia Appendix 3 The top 10 single Chinese herbal medicines for diabetic kidney disease among 173,525 prescriptions.

Multimedia Appendix 4 The top 10 two Chinese herbal medicine combinations for diabetic kidney disease.

Multimedia Appendix 5 Chinese herbal medicines in each cluster.

Multimedia Appendix 6 Examples of the Chinese herbal medicine-ingredient-target protein network.

Multimedia Appendix 7 Differences in the physiochemical characteristics of Chinese herbal medicine (767 ingredients) and Western medicine (37 ingredients) used for diabetic nephropathy.

Multimedia Appendix 8 Physiochemical characteristics of 767 ingredients contained in the Chinese Herbal Medicine Network (CMN) and 37 compounds of Western medicine for diabetic kidney disease. The comparisons between density functions were performed by the two-sample Kolmogorov-Smirnov test.

## Abbreviations

ACEi: angiotensin-converting enzyme inhibitor
aHR: adjusted hazard ratio
API: application programming interface
ARB: angiotensin receptor blocker
ARM: association rule mining
CHM: Chinese herbal medicine
CKD: chronic kidney disease
CMN: Chinese Herbal Medicine Network
DKD: diabetic kidney disease
DM: diabetes mellitus
DN: diabetic nephropathy
DRI: direct renin inhibitor
ESRD: end-stage renal disease
FDR: false discovery rate
GLP-1: glucagon-like peptide-1
HF: herbal formula
HR: hazard ratio
ICD-9-CM: International Classification of Diseases 9, Clinical Modification
IHD: ischemic heart disease
IPTW: inverse probability treatment weighting
NHIRD: National Health Insurance Research Database
NSAID: nonsteroidal anti-inflammatory drug
RAAS: renin-angiotensin-aldosterone system
SGLT2i: sodium-glucose cotransporter 2 inhibitor
SH: single herb
SNA: social network analysis
TCM: Traditional Chinese medicine
WM: Western medicine
