# Supplementary material for: Deciphering the Efficacy and Mechanisms of Chinese Herbal Medicine for Diabetic Kidney Disease by Integrating Web-Based Biochemical Databases and Real-World Clinical Data: Retrospective Cohort Study
Source: JMIR Med Inform. 2021 May 11;9(5):e27614. doi: 10.2196/27614 (PMC8150407; doi:10.2196/27614)
Supplement: Multimedia Appendix 1 [file medinform_v9i5e27614_app1.docx]

| **Multimedia Appendix 1.** Standardized mean differences (%) after inverse probability treatment weighting between Chinese herbal medicine clusters and the Western medicine cohort. | | | | | | | | | | | | | |
| --- | --- | --- | --- | --- | --- | --- | --- | --- | --- | --- | --- | --- | --- |
|  | | Whole  cohort  (n= 20,947) | Cluster 1  (n=5,272) | Cluster 2  (n=2,275) | Cluster 3  (n=2,139) | Cluster 4  (n=905) | Cluster 5  (n=1,144) | Cluster 6  (n=665) | Cluster 7  (n=196) | Cluster 8  (n=336) | Cluster 9  (n=243) | Cluster 10  (n=286) | Cluster 11  (n=196) |
|  | |  |  |  |  |  |  |  |  |  |  |  |  |
| **Gender** | | 2.4 | 2.7 | 4.0 | 2.5 | -3.6 | 1.7 | 2.2 | 7.9 | 2.5 | 3.8 | 3.9 | 2.3 |
| **Age group** | | -0.9 | -1.1 | -1.8 | -1.7 | -1.4 | -4.8 | -4.6 | -3.7 | -0.2 | -0.7 | -2.0 | 3.9 |
| **Co-morbidities** | |  |  |  |  |  |  |  |  |  |  |  |  |
|  | Hypertension | 0.2 | -0.4 | -1.2 | -0.0 | 7.7 | -1.5 | -1.0 | 5.3 | -6.8 | -2.5 | 3.6 | 0.0 |
|  | Hyperlipidemia | 1.7 | 1.6 | 1.9 | 2.4 | 7.8 | 2.9 | 3.3 | 6.4 | 2.0 | 2.4 | -1.3 | 1.1 |
|  | Heart failure | -0.0 | -1.2 | -1.7 | -1.5 | 7.8 | -2.9 | -7.8 | 13.6 | -3.4 | -1.4 | 3.8 | -4.8 |
|  | IHD | -0.4 | -0.9 | -0.4 | 0.3 | 1.2 | -0.8 | -3.1 | 2.3 | -4.2 | -4.5 | -2.9 | 1.5 |
|  | CVD | -0.6 | -2.0 | -0.5 | -1.7 | 3.1 | 0.6 | -0.1 | 3.4 | 2.2 | -0.8 | 9.0 | 0.3 |
|  | Hyperuricemia | 1.3 | 1.1 | 2.0 | 2.4 | 1.6 | 4.4 | -1.8 | 2.5 | -1.1 | -0.4 | 0.8 | 0.7 |
|  | Modified DCSI score | -0.1 | -0.5 | -1.8 | -0.6 | 2.7 | 1.5 | -5.4 | 12.5 | -2.0 | 1.3 | 4.9 | -2.6 |
| **Medications** | |  |  |  |  |  |  |  |  |  |  |  |  |
|  | Diabetic drugs |  |  |  |  |  |  |  |  |  |  |  |  |
|  | Insulin analogs | 0.0 | 0.1 | -2.4 | 0.7 | 10.1 | -0.7 | -3.7 | -0.2 | 4.6 | -3.7 | 1.8 | -2.8 |
|  | OHAs | 1.2 | 0.8 | -0.4 | 0.3 | -0.6 | 0.3 | -0.2 | -3.8 | 0.6 | -2.4 | -0.4 | -2.3 |
|  | Lipid-lowering agent |  |  |  |  |  |  |  |  |  |  |  |  |
|  | Statin/Fibrate | 1.6 | 1.1 | 1.7 | 1.5 | 10.6 | 5.2 | 1.0 | 8.5 | 1.5 | -1.0 | -4.5 | 3.5 |
|  | Anti-hypertensives |  |  |  |  |  |  |  |  |  |  |  |  |
|  | ACEi/ARB | 1.0 | 0.9 | -0.3 | 0.3 | 5.2 | 1.2 | -1.6 | 4.0 | 3.1 | -1.6 | -1.3 | 0.4 |
|  | Others | -0.1 | -0.6 | 0.3 | -0.8 | -2.3 | 0.4 | -3.8 | 7.2 | -5.8 | -0.5 | 5.4 | -4.0 |
|  | Analgesics, aspirin |  |  |  |  |  |  |  |  |  |  |  |  |
|  | NSAIDs | -0.5 | -0.8 | 0.1 | -0.0 | -8.9 | -0.4 | -1.6 | 1.1 | 0.2 | 4.6 | -1.8 | 0.8 |
|  | COX-2 inhibitors | 0.0 | -0.9 | 0.4 | -0.2 | -8.2 | 2.2 | -5.6 | -1.6 | 2.4 | 0.8 | 0.5 | 0.1 |
|  | Acetaminophen | -0.8 | -1.3 | -0.5 | -2.1 | -5.3 | 0.8 | 7.9 | 3.5 | -4.3 | -0.2 | -1.2 | -5.3 |
|  | Aspirin | -0.4 | -0.7 | 0.1 | -0.5 | -5.7 | 1.5 | -4.4 | 3.6 | 1.6 | 2.8 | 6.9 | 2.6 |
| **Insured level** | | 0.8 | 0.7 | 0.5 | -0.1 | -2.5 | 4.1 | 2.3 | -5.7 | 15.3 | 0.2 | 2.3 | -1.6 |
| **Geolocation** | | -0.0 | 0.5 | -0.9 | -0.0 | 0.1 | 1.0 | -0.6 | -1.6 | 6.4 | -2.0 | 9.0 | 2.4 |

Covariates with standardized mean differences less than 10 symbolized no differences between CHM and WM cohorts.
