# Supplementary material for: Deciphering the Efficacy and Mechanisms of Chinese Herbal Medicine for Diabetic Kidney Disease by Integrating Web-Based Biochemical Databases and Real-World Clinical Data: Retrospective Cohort Study
Source: JMIR Med Inform. 2021 May 11;9(5):e27614. doi: 10.2196/27614 (PMC8150407; doi:10.2196/27614)
Supplement: Multimedia Appendix 2 [file medinform_v9i5e27614_app2.docx]

| **Multimedia Appendix 2.** Sensitivity analyses on the risks of mortality among Chinese herbal medicine users. | | |
| --- | --- | --- |
| Model | aHR (99% CI) | *P* |
|  |  |  |
| Propensity score with 1:1 matching (n=41,894) | 0.41 (0.39-0.44) | <.001 |
| Excluding DKD patients with CHM use before diagnosis of DKD (n=141,196) | 0.45 (0.42-0.48) | <.001 |
| Excluding late CHM users, who started using CHM for DKD within 180 days before death (n=152,175) | 0.38 (0.36-0.40) | <.001 |
| Excluding ESRD patients (n=135,380) | 0.38 (0.36-0.41) | <.001 |
| ESRD-only patients (n=16,977) | 0.45 (0.41-0.50) | <.001 |
|  | | |
