# Supplementary material for: Deciphering the Efficacy and Mechanisms of Chinese Herbal Medicine for Diabetic Kidney Disease by Integrating Web-Based Biochemical Databases and Real-World Clinical Data: Retrospective Cohort Study
Source: JMIR Med Inform. 2021 May 11;9(5):e27614. doi: 10.2196/27614 (PMC8150407; doi:10.2196/27614)
Supplement: Multimedia Appendix 3 [file medinform_v9i5e27614_app3.docx]

| **Multimedia Appendix 3.** The top 10 single Chinese herbal medicines for diabetic kidney disease among 173,525 prescriptions. | | |
| --- | --- | --- |
| CHM | Counts | Prevalence (%) |
|  |  |  |
| Ji-Sheng-Shen-Qi-Wan | 39,642 | 22.85 |
| Liu-Wei-Di-Huang-Wan | 21,466 | 12.37 |
| *Salvia miltiorrhiza* Bge. | 19,770 | 11.39 |
| *Plantago asiatica* L. | 16,071 | 9.26 |
| *Astragalus membranaceus* (Fisch.) Bge. or *Astragalus mongholicus* Bge. | 15,791 | 9.1 |
| *Rheum palmatum* L. or *Rheum tanguticum* Maxim. ex Balf. or *Rheum officinale* Baill. | 14,953 | 8.62 |
| Zhu-Ling-Tang | 14,558 | 8.39 |
| Zhi-Bai-Di-Huang-Wan | 12,737 | 7.34 |
| *Achyrantes bidentata* BL. | 12,393 | 7.14 |
| Ba-Wei-Di-Huang-Wan | 11,630 | 6.7 |
