# Supplementary material for: Deciphering the Efficacy and Mechanisms of Chinese Herbal Medicine for Diabetic Kidney Disease by Integrating Web-Based Biochemical Databases and Real-World Clinical Data: Retrospective Cohort Study
Source: JMIR Med Inform. 2021 May 11;9(5):e27614. doi: 10.2196/27614 (PMC8150407; doi:10.2196/27614)
Supplement: Multimedia Appendix 4 [file medinform_v9i5e27614_app4.docx]

| **Multimedia Appendix 4.** The top 10 two Chinese herbal medicine combinations for diabetic kidney disease. | | | | |
| --- | --- | --- | --- | --- |
| CHM A | CHM B | Prevalence (%) | Confidence | Lift |
|  |  |  |  |  |
| Wu-Lin-San | Ji-Sheng-Shen-Qi-Wan | 4.8 | 32.6 | 1.4 |
| *Salvia miltiorrhiza* Bge. | Ji-Sheng-Shen-Qi-Wan | 4.6 | 24.5 | 1.1 |
| Zhu-Ling-Tang | Ji-Sheng-Shen-Qi-Wan | 4.4 | 28.8 | 1.3 |
| *Plantago asiatica* L. | *Achyrantes bidentata* BL. | 4.3 | 25.8 | 3.1 |
| *Astragalus membranaceus* (Fisch.) Bge. or *Astragalus mongholicus* Bge. | Ji-Sheng-Shen-Qi-Wan | 3.8 | 27 | 1.2 |
| Wu-Lin-San | *Plantago asiatica* L. | 3.3 | 21.7 | 2.4 |
| *Eucommia ulmoides* Oliv. | Ji-Sheng-Shen-Qi-Wan | 2.9 | 24.6 | 1.1 |
| *Dipsacus asperoides* C. Y. Cheng at T. M. Ai | *Eucommia ulmoides* Oliv. | 2.7 | 42.2 | 6.8 |
| Bei-Xie-Fen-Qing-Yin | Ji-Sheng-Shen-Qi-Wan | 2.2 | 24.5 | 1.1 |
| *Panax notoginseng* (Burk.) F. H. Chen | *Salvia miltiorrhiza* Bge. | 2.1 | 40.3 | 3.6 |

Prevalence was the percentage of each combination of all prescriptions (n=173,525). Confidence and lift presented the strength of each combinations, and higher value represented stronger connections between CHMs.
