# Supplementary material for: Deciphering the Efficacy and Mechanisms of Chinese Herbal Medicine for Diabetic Kidney Disease by Integrating Web-Based Biochemical Databases and Real-World Clinical Data: Retrospective Cohort Study
Source: JMIR Med Inform. 2021 May 11;9(5):e27614. doi: 10.2196/27614 (PMC8150407; doi:10.2196/27614)
Supplement: Multimedia Appendix 5 [file medinform_v9i5e27614_app5.docx]

| **Multimedia Appendix 5.** Chinese herbal medicines in each cluster. | |
| --- | --- |
| Cluster number | CHM |
|  |  |
| 1 | Ji-Sheng-Shen-Qi-Wan, Zhu-Ling-Tang, Wu-Lin-San, *Plantago depressa* Willd. (Fam. Plantaginaceae), Bei-Xie-Fen-Qing-Yin, *Imperata cylindrica* Beauv. var. major (Nees) C. E. Hubb. (Fam. Gramineae), *Zea mays* L., Ma-Zi-Ren-Wan, *Rubus chingii* Hu (Fam. Rosaceae), Yu-Quan-Wan, Bu-Zhong-Yi-Qi-Tang, *Dioscorea septemloba* Thunbt or *Dioscorea hypoglauca* Palibin, *Alpinia oxyphylla* Miq. (Fam. Zingiberaceae), Sheng-Mai-San, *Alisma orientale* Samuels (Fam. Alismataceae), Sang-Piao-Xiao-San, *Coix lacryma-jobi* L. var. ma-yuen (Roman.) Stapf, Zhi-Gan-Cao-Tang, Dao-Shui-Fu-Ling-Tang, *Lindera aggregata* (Sims) Kosterm. (Fam. Lauraceae), *Desmodium styracifolium* (Osb.) Merr. (Fam. Fabaceae) |
| 2 | *Astragalus membranaceus* (Fisch. ) Bge. *Astragalus mongholicus* Bge. (Fam. Fabaceae), *Dioscorea opposita* Thunb. (Fam. Dioscoreaceae), *Phellodendron amurense* Rupr. (Fam. Rutaceae), *Anemarrhena asphodeloides* Bge. (Fam. Liliaceae), *Angelica sinensis* (Oliv.) Diels. (Fam. Apiaceae), *Codonopsis tangshen* Oliv. (Fam.Campanulaceae), *Leonurus heterophyllus* Sweet (Fam. Lamiaceae), *Atractylodes macrocephala* Koidz. (Fam. Asteracae), *Poria cocos* (Schw.) Wolf (Fam. Polyporaceae), *Euryale ferox* Salisb. (Fam. Nymphaeaceae), Qing-Xin-Lian-Zi-Yin, *Rheum officinale* Baill. (Fam. Polygonaceae) |
| 3 | *Salvia miltiorrhiza* Bge. (Fam. Lamiaceae), *Panax notoginseng* (Burk.) F. H. Chen (Fam. Araliaceae), *Pueraria thomsonii* Benth. (Fam. Fabaceae), *Trichosanthes japonica* Regel (Fam. Curcubitaceae), *Ophiopogon japonicus* Ker-Gawl. (Fam. Liliaceae), *Schisandra chinensis* (Turcz.) Baill. (Fam. Magnoliaceae), *Dendrobium nobile* Lindl. (Fam. Orchidaceae), *Atractylodes chinensis* DC. Koidz. (Fam. Asteraceae), *Scrophularia ningpoensis* Hemsl. (Fam. Scrophulariaceae), *Crataegus pinnatifida* Bge. (Fam. Rosaceae), *Ligusticum chuanxiong* Hort. (Fam. Apiaceae) |
| 4 | *Epimedium sagittatum* (Sieb. et Zucc.) Maxim. (Fam. Berberidaceae), *Morinda officinalis* How. (Fam. Rubiaceae), Huan-Shao-Dan, *Cistanche deserticola* Y. C. Ma (Fam. Orobanchaceae), *Cuscuta chinensis* Lam.Cuscuta japonica Choisy (Fam. Convolvulaceae), Jin-Suo-Gu-Jing-Wan, *Cynomorium songaricum* Rupr. (Fam. Cynomoriaceae), Wu-Pi-Yin, You-Gui-Wan |
| 5 | *Achyrantes bidentata* BL. (Fam. Amaranthaceae), *Eucommia ulmoides* Oliv. (Fam. Eucommiaceae), *Dipsacus asperoides* C. Y. Cheng at T. M. Ai (Fam. Dipsacaceae), Liu-Wei-Di-Huang-Wan, *Chaenomeles speciosa* (Sweet) Nakai (Fam. Rosaceae) |
| 6 | *Zingiber officinale* Rosc. (Fam. Zingiberaceae), *Aconitum carmichaelii*, *Asarum sieboldii* Miq. (Fam. Aristolochiaceae), *Cinnamomum cassia* Blume (Fam. Lauraceae) |
| 7 | Bu-Yang-Huan-Wu-Tang, Jia-Wei-Xiao-Yao-San |
| 8 | Shu-Jing-Hwo-Shiee-Tang, Du-Huo-Ji-Sheng-Tang |
| 9 | *Eclipta prostrata* L. (Fam. Asteraceae), *Ligustrum lucidum* Ait. (Fam. Oleaceae) |
| 10 | *Commiphora myrrha* Engl. (Fam. Burseraceae), *Boswellia carterii* Birdw. (Fam. Burseraceae) |
| 11 | *Elephantopus mollis* H. B. K., *Ludwigia octovalvis* |
