# Supplementary material for: Deciphering the Efficacy and Mechanisms of Chinese Herbal Medicine for Diabetic Kidney Disease by Integrating Web-Based Biochemical Databases and Real-World Clinical Data: Retrospective Cohort Study
Source: JMIR Med Inform. 2021 May 11;9(5):e27614. doi: 10.2196/27614 (PMC8150407; doi:10.2196/27614)
Supplement: Multimedia Appendix 6 [file medinform_v9i5e27614_app6.docx]

| **Multimedia Appendix 6.** Examples of the Chinese herbal medicine-ingredient-target protein network. | | | |
| --- | --- | --- | --- |
| CHM | Ingredients | Target protein | Score |
|  |  |  |  |
| *Salvia miltiorrhiza* Bge. | Tanshinone I | CCND1  NOS3  CP1A2 | 0.800  0.830  0.968 |
|  | Tanshinone IIA | MMP2  CCND1  NR0B2  ATF3  PTN3 | 0.957  0.800  0.800  0.800  0.800 |
|  | Salvianolic acid B | IL2  IL15  MK08 | 0.800  0.800  0.800 |
| *Astragalus membranaceus* (Fisch.) Bge. or *Astragalus mongholicus* Bge. | Astragaloside IV | PAI1  CP2C9  P53  AKT1  ACE  TLR4  F6X3S4 | 0.800  0.800  0.800  0.800  0.800  0.800  0.800 |
|  | Calycosin | BLVRB  HMGB1 | 0.830  0.830 |
|  | Flavaxin | PYGL  S52A3  PPA5  BLVRB  PPAT  PPAC  PPAP  PPA6  RIFK | 0.958  0.900  0.939  0.989  0.800  0.800  0.800  0.800  1.000 |

Scores indicated the strength of relations between ingredients and target proteins acquired from the web-based STITCH database via application programming interface (API). A higher score represented higher confidence in ingredient-target proteins connections.
