# Supplementary material for: Deciphering the Efficacy and Mechanisms of Chinese Herbal Medicine for Diabetic Kidney Disease by Integrating Web-Based Biochemical Databases and Real-World Clinical Data: Retrospective Cohort Study
Source: JMIR Med Inform. 2021 May 11;9(5):e27614. doi: 10.2196/27614 (PMC8150407; doi:10.2196/27614)
Supplement: Multimedia Appendix 7 [file medinform_v9i5e27614_app7.docx]

| **Multimedia Appendix 7.** Differences in the physiochemical characteristics of Chinese herbal medicine (767 ingredients) and Western medicine (37 ingredients) used for diabetic nephropathy. | | | | | |
| --- | --- | --- | --- | --- | --- |
|  | CHM | | WM | | *P* |
|  |  |  |  |  |  |
| Molecular Weight | 245.3 | (152.1-350.0) | 435.5 | (414.0-498.6) | <.001 |
| Aromatic Bonds Count | 6.0 | (0.0-12.0) | 12.0 | (6.0-22.5) | <.001 |
| Hydrogen Bond Acceptors | 2.0 | (1.0-4.0) | 7.0 | (6.0-7.0) | <.001 |
| Hydrogen Bond Donors | 1.9 | (1.0-3.0) | 2.0 | (1.0-2.0) | .20 |
| Rotatable Bonds Count | 6.0 | (4.0-11.7) | 13.0 | (11.5-15.0) | <.001 |
| Topological Polar Surface Area | 62.1 | (37.3-104.5) | 112.1 | (97.8-145.7) | <.001 |
| XLogP | 2.0 | (-0.2-3.8) | 1.6 | (0.4-3.1) | .14 |

*Statistics were presented as median (25^th^-75^th^ percentile) and calculated by the Mann-Whitney U test
