# Supplementary figures and images for: Deciphering the Efficacy and Mechanisms of Chinese Herbal Medicine for Diabetic Kidney Disease by Integrating Web-Based Biochemical Databases and Real-World Clinical Data: Retrospective Cohort Study
Source: JMIR Med Inform. 2021 May 11;9(5):e27614. doi: 10.2196/27614 (PMC8150407; doi:10.2196/27614)

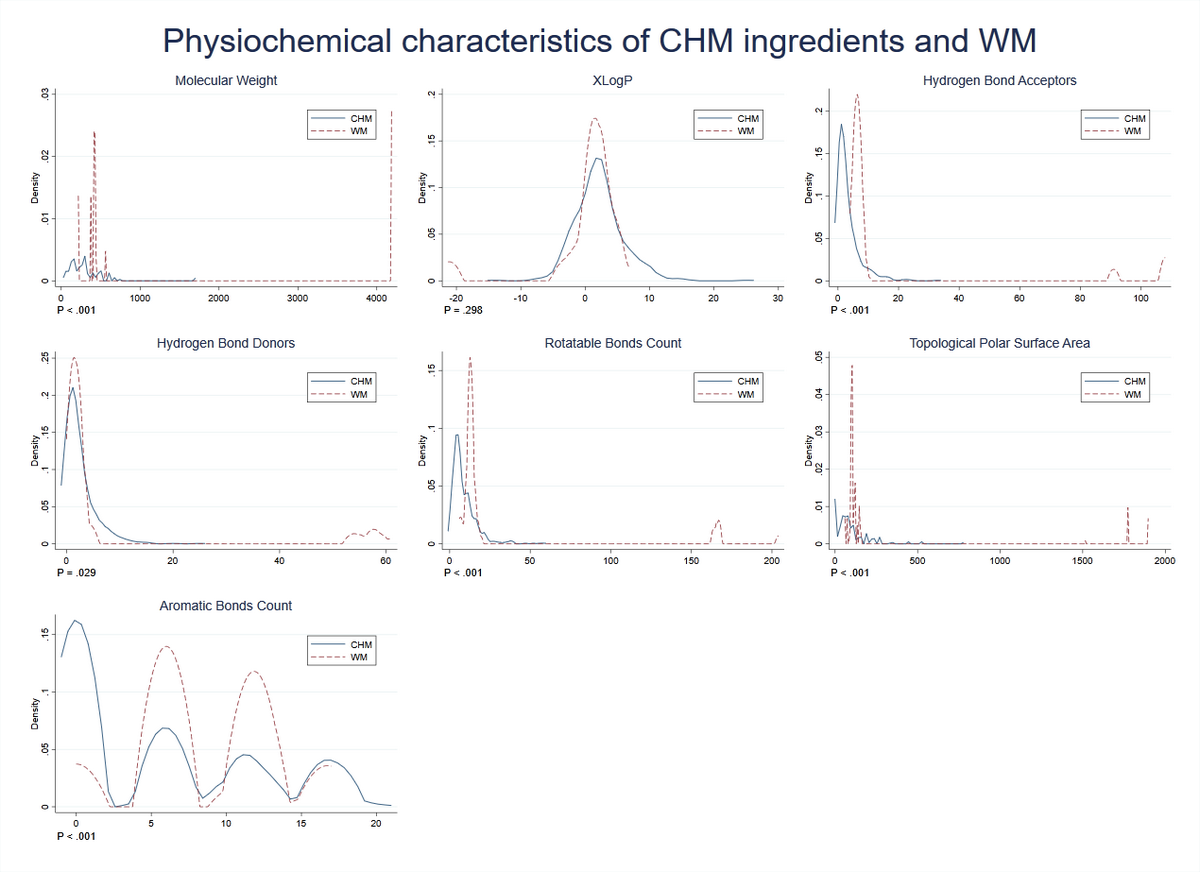

Supplement: Multimedia Appendix 8 [file medinform_v9i5e27614_app8.png]
